# Gender Differences in the Psychopharmacological Treatment of Forensic In-Patients With Schizophrenia

**DOI:** 10.3389/fpsyt.2022.907123

**Published:** 2022-07-15

**Authors:** Juliane Mayer, Nenad Vasic, Viviane Wolf, Ivonne Steiner, Verena Klein, Michael Fritz, Philipp Rothe, Judith Streb, Manuela Dudeck

**Affiliations:** ^1^Department of Forensic Psychiatry and Psychotherapy, kbo-Isar-Amper-Clinic Taufkirchen (Vils), Taufkirchen, Germany; ^2^Clinic for Psychiatry and Psychotherapy, Clinic Centre Christophsbad, Göppingen, Germany; ^3^Department of Psychiatry and Psychotherapy, Medical Faculty, LVR-Clinic Düsseldorf, Heinrich Heine University Düsseldorf, Düsseldorf, Germany; ^4^Department of Forensic Psychiatry and Psychotherapy, District Hospital Günzburg, Ulm University, Günzburg, Germany; ^5^School of Health and Social Sciences, AKAD University of Applied Sciences, Stuttgart, Germany; ^6^kbo-Lech-Mangfall-Clinic Agatharied, Hausham, Germany

**Keywords:** gender differences, schizophrenia, psychopharmacotherapy, antipsychotics, female offenders, forensic psychiatry

## Abstract

**Background:**

In forensic psychiatry, psychopharmacological treatment plays a crucial role for patients with schizophrenia in improving their medical as well as legal prognosis. However, an increase in the number of females entering forensic treatment has yet to yield empirical research on the outcome of psychopharmacological treatment of female patients with schizophrenia in terms of efficacy and tolerability.

**Aims:**

The aim of the present study is to elucidate pharmacological treatment strategies of women with schizophrenia in forensic psychiatry in comparison with men.

**Methods:**

This study compares psychopharmacological treatment strategies, psychopathological features, as well as neurological and metabolic side effects of treatment between 29 female and 29 male in-patients with schizophrenia in three forensic facilities in Bavaria, Germany.

**Results:**

Results show significant differences between genders. Poorer psychopathological and neurological features were found in the female sample, while men registered worse metabolic parameters. In terms of psychopharmacological treatment strategies, female in-patients were more often prescribed second-generation depot antipsychotics. Surprisingly, the potency of the dosages did not differ between genders. The results suggest that female forensic patients with schizophrenia have more severe and refractory diseases than their male counterparts.

**Conclusion:**

Recommendations for gender-specific treatment strategies are derived.

## Introduction

Schizophrenia is one of the most severe, diverse, and detrimental psychiatric disorders, accompanied by major psychological, social, and cognitive impairments. Treatment guidelines consistently recommend the administration of antipsychotic medication as a fundamental component of a multimodal treatment framework (1). Since currently available antipsychotics differ in their efficacy and tolerability (2), clinicians are advised to not only consider the ability of the particular antipsychotic and dosage to reduce individual negative and positive schizophrenic symptoms in order to improve psychopathological features (3), but to also take into account the typical adverse side effects profile of the drug to avoid or reduce their occurrence. In particular, neurological and metabolic side effects appear to be the best studied and documented when it comes to antipsychotic medication (4).

Ensuring effective antipsychotic treatment for patients with schizophrenia is of particular importance with regard to forensic psychiatry. While schizophrenia patients have an increased risk of delinquency and violent behavior compared to the general population (5), psychopharmacological treatment is not only focused on the remittance of symptoms, but also the reduction of aggressive and criminal behavior in order to improve legal prognosis.

Evidence regarding psychopharmacological treatment strategies for schizophrenia in forensic settings is rare, focusing mainly on differences between general psychiatric and forensic samples (6, 7). Vasic et al. (8) not only examined the prescribed medication, but also the respective psychopathological status as well as neurological and metabolic side effects of in-patients with schizophrenia in either forensic (*n* = 29) or general psychiatry (*n* = 31) in two southern German clinics. They found that forensic patients received dosages of higher neuroleptic potency, albeit fewer psychopharmaceuticals. While there were no significant differences for neurological impairments or metabolic side effects, forensic patients exhibited more pronounced psychopathological features of delusions of grandeur, animosity, flattening of affect, week will, social passivity, apathy, uncooperative behavior, and poor impulse control, indicating that paranoid and negative symptoms predominate in the forensic patients.

Previous research on psychopharmacological treatment of forensic patients with schizophrenia has mainly been focused on male subjects. With the actual rate of delinquent behavior among schizophrenia patients amounting to 13.5% (9), men (10.7%) are significantly more affected than women (2.7%) (10). However, an increase in the number of females hospitalized in forensic psychiatry has been observed in many countries, urging the need for evidence-based research on gender-specific treatment strategies for female forensic patients (11). To our knowledge, only one study directly compares treatment characteristics of male and female forensic patients with schizophrenia. Günther et al. (12) used a latent class analysis with 31 women and 329 men to show that the female-dominated class was equally likely to receive high antipsychotic dosages and was less likely to benefit from in-patient forensic treatment. Alas, they did not further elaborate on the specific psychopharmacological treatment strategies or examine possible side effects of the medication. However, previous research in non-forensic settings reported significant gender differences in schizophrenia patients not only regarding clinical, social, and illness course characteristics, but also the prescription of antipsychotic medication. In general, women were found to show a superior response rate to antipsychotic medication, to need lower doses, especially pre-menopause, and to exhibit better social functioning and outcome, while man were prone to more substance abuse (13–15). Regarding metabolic and neurological side effects, women have shown to be at greater risk for metabolic complications (13, 16) and are generally more frequently affected by extrapyramidal side effects than men (17). When it comes to forensic samples, however, gender differences in psychopathology, especially concerning proneness for violent behavior, seem to diminish (12), suggesting that female forensic patients with schizophrenia are quite similar to their male counterparts in that respect.

The aim of this exploratory study is to elucidate pharmacological treatment strategies of women with schizophrenia in forensic psychiatry in comparison with men. In order to better understand gender-specific treatment needs and practice of the understudied subgroup of female offenders with schizophrenia, we are especially interested in uncovering potential gender differences in the relationships between the targeted psychopathological symptoms and drug therapy as well as in the occurrence of side effects. Therefore, we expand the research design by Vasic et al. (8) to include a female forensic sample. Particularly, we aim to examine gender differences between the male forensic sample previously studied by Vasic et al. (8) and a corresponding female forensic sample pertaining to medication and dosage, psychopathological status, as well as neurological and metabolic side effects to be able to assess pharmacological treatment strategies in terms of both their efficacy and tolerability and, ultimately, improve gender-specific treatment of female forensic patients with schizophrenia.

## Materials and Methods

### Participants

We studied 29 female forensic in-patients being treated in the Department for Forensic Psychiatry and Psychotherapy of the kbo-Isar-Amper-Klinikum Taufkirchen (Vils), Germany, and 29 male forensic in-patients being treated in the Department of Forensic Psychiatry and Psychotherapy at the District Hospital Günzburg, Germany, and in the Department for Forensic Psychiatry and Psychotherapy at the District Hospital Kaufbeuren, Germany [see also Vasic et al. (8)]. All participants were clinically diagnosed with a schizophrenia spectrum disorder (F2) according to ICD-10. The female patients were recruited between November 2018 and May 2019 on a total of five both closed and open wards. The male patients were recruited July 2014 through October 2014 on a total of four wards. Most patients were hospitalized under the terms of a hospital treatment order according to Section 63 of the German Criminal Code, while eleven were court ordered to a provisional placement according to Section 126a of the German Code of Criminal Procedure and two patients were hospitalized under the terms of an addiction treatment according to Section 64 of the German Criminal Code. Table 1 shows group characteristics.

**TABLE 1:** Group characteristics of male and female in-patients with schizophrenia.

	Men (*n* = 29)	Women (*n* = 29)		
		
	*M* (SD)/ prevalence in %	*M* (SD)/ prevalence in %	*U/χ* ^2^	Significance level (*p*)
Age (years)	41.0 (9.3)	43.9 (12.4)	365.0^a^	0.388
**Education level**				
Education to end of 9th grade (“Hauptschule”)	75.9%	44.8%	11.510^b^	0.021*
Education to end of 9th grade (“Realschule”)	17.2%	27.6%		
Education to end of 12th or 13th grade (“Abitur”) and higher education	6.9%	27.8%		
Age of onset (years)	27.3 (8.4)	28.5 (12.3)	397.5^a^	0.892
Duration of illness (years)	13.6 (6.2)	16.0 (11.1)	366.5^a^	0.528
Number of hospitalizations	7.9 (9.0)	7.5 (7.5)	413.0^a^	0.907
Body mass index	26.6 (7.1)	27.8 (6.4)	405.0^a^	0.987
**Family status**				
Single	89.7%	58.6%	10.284^b^	0.016*
Married	3.4%	10.3%		
Divorced/widowed	6.9%	31.0%		
**Profession**				
Untrained	37.9%	58.6%		
Trained worker	48.3%	17.2%	6.367^b^	0.041*
Employee	13.8%	24.1%		
**Family psychiatric history**				
Schizophrenia	50.0%	20.7%	8.996^b^	0.011*
Other psychiatric disorders	3.8%	31.0%		
Age at first imprisonment (years)	31.05 (9.7)	38.1 (12.0)	209.0^a^	0.036*
**Offense characteristics**				
Property offense	24.1%	3.4%	6.078^b^	0.014*
Traffic offense	13.8%	0.0%	4.811^b^	0.028*
Arson	6.9%	27.6%	3.881^b^	0.049*
Offense committed under the influence of a substance	6.9%	41.4%	8.199^b^	0.004**

*M, mean; SD, standard deviation.*

*^a^Mann–Whitney-U.*

*^b^Chi-square χ^2^ (Pearson).*

*p < 0.05, **p < 0.01 (asymptotic, two-tailed).

### Procedure

The project had been approved by the local ethics committee (Ulm University, Germany). Patients were informed about the study objectives and provided written informed consent, receiving neither financial nor non-financial compensation for their participation. Collection of data from patient files and completion of the questionnaires was conducted by experienced research assistants working in the institutions. Collection of laboratory data and physical examination was performed by clinicians who treated the particular patients.

### Measures

The data was recorded using a self-designed data entry form. We examined patients in person as well as their medical files and official court records to collect the following data: age, gender, family status, education level, profession, main diagnosis and secondary diagnosis, age at first hospitalization, number of hospitalizations, suicidal acts and self-harm in medical history, mental disorders in the family history, past substance abuse, age at first imprisonment, age at first conviction, regulatory framework of the current hospitalization, duration of the current hospitalization, characteristics of the offense leading to the current hospitalization (violent offense, e.g., homicide/manslaughter, robbery, and assault; property offense; arson; sexual offense; traffic offense; drug-related offense; whether the offense was committed under the influence of a substance), as well as current medication including dosage and form of application. For the female sample, we also assessed menopausal status.

To evaluate the effectiveness of the antipsychotic medication, we assessed the current psychopathological status using the Positive and Negative Syndrome Scale (PANSS) (18) and the Brief Psychiatric Rating Scale (BPRS) (19). While the BPRS measures psychiatric symptoms like depression, anxiety, and psychotic symptoms in general, the PANSS specifically targets symptom severity of patients with schizophrenia. The diagnostic criteria for the deficit syndrome of schizophrenia (DSS) (20, 21) were used for a closer examination of the prevalence and severity of negative schizophrenic symptoms. Finally, the Global Assessment Scale (GAS) (22) was used to assess a patient’s overall level of social and psychological functioning.

To account for possible side effects of the antipsychotic medication, we examined metabolic and neurological abnormalities. Thus, specific metabolic laboratory parameters, i.e., glycated hemoglobin (HBa1c), cholesterol, triglycerides, C-reactive protein (CRP), gamma-glutamyltransferase (GGT), and glutamic oxaloacetic transaminase (GOT), were retrieved. In addition, height and weight at time of admission and examination were recorded as well as waist and hip measurements were taken in order to calculate the body mass index (BMI) and waist-to-hip ratio to evaluate obesity-associated metabolic complications. Neurological side effects were examined using the Extrapyramidal Symptom Scale (EPS) (23) to rate extrapyramidal movement disorders, the Barnes Akathisia Scale (BAS) (24) to assess drug-induced restlessness, and the Abnormal Involuntary Movement Scale (AIMS) (25) describing dyskinesia associated with antipsychotic medication.

### Data Analysis

Data analysis was performed with the Statistical Package for Social Sciences [SPSS Statistics for Windows; IBM Corp., (26)]. First, we calculated absolute and relative frequencies, mean values, and standard deviations separately for both genders. Since most variables did not meet conditions for normal distribution or variance homogeneity, group comparisons were performed using the Mann–Whitney-*U* test, while the Pearson Chi-square (χ^2^) independence test was used to compare frequencies.

## Results

### Group Characteristics

A comparison of the male and female in-patients participating in this study (Table 1) revealed significant differences regarding their social situation, i.e., education level, profession, and family status. The women had a higher level of education overall and were more likely to have been married before hospitalization, while men were more likely to have completed their occupational training.

Regarding clinical characteristics, schizophrenia was significantly more prevalent in the family history of men, while women more often reported other mental illnesses in their families. However, age of onset, number of prior hospitalizations, and duration of illness did not differ between genders.

Differences were also found with regard to criminal aspects. Age at first imprisonment was significantly lower for men than for women. The distribution of the offenses leading to the hospitalization also showed significant differences. Women more frequently committed arson and were more frequently under the influence of substances during the act. Men, on the other hand, committed property and traffic offenses more frequently. The samples did not differ with regard to violent and sexual delinquency as well as drug-related crime.

### Psychopathological Features

Table 2 depicts significant differences between genders in their psychopathological status as assessed with the measures applied in this study. Overall, a poorer psychopathological status was attributed to women compared to men. Women suffered more from general psychopathological symptoms such as concerns about somatic health, guilt, anxiety, and depression. They showed higher levels of suspiciousness and more often stereotyped thoughts as well as lack of judgment and insight. However, only differences in feelings of guilt as measured by the BPRS and PANSS persisted after Bonferroni correction.

**TABLE 2:** Differences in the psychopathological features between male and female forensic in-patients with schizophrenia.

	Men (*n* = 29)	Women (*n* = 29)		
		
	*M* (SD)/ prevalence in %	*M* (SD)/ prevalence in %	*U/χ* ^2^	Significance level (*p*)
**BPRS**				
Somatic concern	2.0 (1.3)	3.1 (1.9)	288.5^a^	0.032*
Anxiety	1.8 (1.2)	3.3 (1.9)	231.0^a^	0.002**
Guilt	1.6 (1.1)	3.1 (1.9)	220.0^a^	0.001***
Total	37.7 (9.6)	43.7 (12.1)	312.5^a^	0.093
**DSS**				
Negative symptoms secondary to drug effects	42.9%	92.0%	14.222^b^	0.000***
GAS	5.03 (1.5)	4.55 (1.5)	337.0^a^	0.182
**PANSS**				
Suspiciousness (P6)	2.0 (1.1)	3.1 (1.7)	264.0^a^	0.012*
Stereotyped thinking (N7)	1.7 (0.8)	2.6 (1.1)	215.5^a^	0.001***
Anxiety (G2)	1.6 (0.8)	2.8 (1.9)	262.5^a^	0.009**
Guilt feelings (G3)	1.7 (1.2)	3.0 (1.7)	222.5^a^	0.001***
Depression (G6)	1.6 (0.7)	2.7 (1.6)	242.5^a^	0.004**
Lack of judgment and insight (G12)	2.5 (1.6)	3.5 (1.5)	266.0^a^	0.014*
Total general psychopathology scale (G)	30.3 (7.6)	38.2 (10.3)	230.5^a^	0.003**
Total	60.7 (16.9)	72.3 (20.1)	286.0^a^	0.036*

*M, mean; SD, standard deviation; BPRS, Brief Psychiatric Rating Scale; DSS, diagnostic criteria for the deficit syndrome of schizophrenia; GAS, Global Assessment Scale; PANSS, Positive and Negative Syndrome Scale.*

*^a^Mann–Whitney-U.*

*^b^Chi-square χ^2^ (Pearson).*

*p < 0.05, **p < 0.01, ***p < 0.001 (asymptotic, two-tailed).

With regard to the assessment of the DSS, negative symptoms shown in the female sample were more likely to be attributed to the effects of the medication. This difference also persisted after Bonferroni correction. No significant differences were found regarding the global assessment of the psychological and social functioning (GAS).

### Neurological Characteristics

Significant group differences in neurological status were found in relation to AIMS, i.e., muscles of facial expression, lips and perioral area, current problems with teeth and/or dentures, as well as extrapyramidal disorders (EPS), i.e., glabella reflex (see Table 3), with women receiving higher ratings for their occurrence or severity. The group difference for muscles of facial expressions as measured by the AIMS persisted even after Bonferroni correction. The results indicate that women in forensic psychiatry with a diagnosis of schizophrenia are significantly more affected by impaired neurological functioning of the face and oral area than men. However, no gender differences were found for symptoms of akathisia (BAS).

**TABLE 3:** Differences in the neurological characteristics between male and female forensic in-patients with schizophrenia.

	Men (*n* = 29)	Women (*n* = 29)		
		
	*M* (SD)/ prevalence in %	*M* (SD)/ prevalence in %	*U/χ* ^2^	Significance level (*p*)
**AIMS**				
Muscles of facial expression	0.2 (0.5)	1.1 (1.2)	204.5^a^	0.000***
Lips and perioral area	0.6 (1.1)	1.1 (1.2)	285.5^a^	0.020*
Current problems with teeth and/or dentures	24.1%	58.6%	7.108^b^	0.008**
**EPS**				
Glabella reflex	1.1 (0.4)	1.5 (0.9)	265.0^a^	0.005**

*M, mean; SD, standard deviation; AIMS, Abnormal Involuntary Movement Scale; EPS, Extrapyramidal Symptom Scale.*

*^a^Mann–Whitney-U.*

*^b^Chi-square χ^2^ (Pearson).*

*p < 0.05, **p < 0.01, ***p < 0.001 (asymptotic, two-tailed).

### Metabolic Parameters

Metabolic parameters showed significant differences between genders for blood serum lipids (see Table 4). Men had higher cholesterol and triglycerides levels than women. These differences were also evident when interpreting the levels according to reference intervals, with borderline or pathological levels for cholesterol and triglycerides being measured significantly more frequently for men than for women. After Bonferroni correction, differences still persisted for cholesterol levels as well as the reference interval for triglycerides. Other blood serum levels or measurements of metabolic parameters (e.g. waist size; change in BMI over time) showed no significant differences.

**TABLE 4:** Differences in blood serum lipids between male and female forensic in-patients with schizophrenia.

	Men (*n* = 29)	Women (*n* = 29)		
		
	*M* (SD)/ prevalence in %	*M* (SD)/ prevalence in %	*U/χ* ^2^	Significance level (*p*)
Cholesterol (mg/dl)	221.5 (43.5)	188.6 (38.7)	131.5^a^	0.004**
**Cholesterol reference interval**				
Normal	35.0%	75.0%	7.886^b^	0.019*
Borderline	5.0%	3.6%		
Pathological	60.0%	21.4%		
Triglycerides (mg/dl)	254.8 (202.9)	119.1 (62.7)	142.0^a^	0.004**
**Triglycerides reference interval**				
Normal	40.0%	89.3%	13.367^b^	0.001**
Borderline	5.0%	0.0%		
Pathological	45.0%	10.7%		

*M, mean; SD, standard deviation.*

*^a^Mann–Whitney-U.*

*^b^Chi-square χ^2^ (Pearson).*

*p < 0.05, **p < 0.01 (asymptotic, two-tailed).

### Psychopharmacological Treatment Strategies

With regard to the total number of drugs, psychopharmaceuticals, and depot preparations prescribed, there were no significant differences between men and women (see Table 5). The same applies to the total number of antipsychotics, benzodiazepines, antidepressants, and anticonvulsants. Significant differences in prescription rates for first-generation (FGA) or second-generation antipsychotics (SGA) were only found for SGA depot preparations, which were significantly more frequently prescribed to women. Comparing chlorpromazine and olanzapine equivalents (27) revealed no differences in the neuroleptic potency of the prescribed antipsychotic drugs between genders. On average, women received dosages of the same potency as men. In the female sample, no significant correlation for the menopausal status and the potency of the dosages was found (*r* = 0.073, *p* = 0.721).

**TABLE 5:** Prescription rates of psychopharmaceuticals in male and female forensic in-patients with schizophrenia, separated into first-generation antipsychotics (FGAs) and second-generation antipsychotics (SGAs) as well as oral and depot preparations.

	Men (*n* = 29)	Women (*n* = 29)		
		
	*M* (SD)/ prevalence in %	*M* (SD)/ prevalence in %	*U*/χ^2^	Significance level (*p*)
Chlorpromazine/ olanzapine equivalents (oral and depot)	587.0 (305.3)/19.6 (10.2)	580.4 (267.7)/19.3 (8.9)	411.0^a^	0.882
Drugs total			7.506^b^	0.585
1–2	51.7%	34.5%		
3–4	34.5%	44.8%		
>4	13.8%	20.7%		
Psychopharmaceuticals total			1.303^b^	0.861
1–2	62.1%	62.1%		
3–4	34.5%	34.5%		
>4	3.4%	3.4%		
FGAs total			0.167^b^	0.920
0	69.0%	72.4%		
1	17.2%	17.2%		
>1	13.8%	10.2%		
FGAs oral			2.791^b^	0.248
0	75.9%	86.2%		
1	24.1%	10.3%		
>1	0.0%	3.4%		
FGAs depot	20.7%	20.7%	0.000^b^	1.000
SGAs total			2.044^b^	0.563
0	10.3%	10.3%		
1	55.2%	69.0%		
>1	34.5%	20.7%		
SGAs oral			4.889^b^	0.180
0	20.7%	44.8%		
1	55.2%	44.8%		
>1	24.1%	10.4%		
SGAs depot	13.8%	44.8%	6.740^b^	0.009**
Depot total	34.5%	62.0%	4.948^b^	0.084

*M, mean; SD, standard deviation; FGAs, first-generation antipsychotics; SGAs, second-generation antipsychotics.*

*^a^Mann–Whitney-U.*

*^b^Chi-square χ^2^ (Pearson).*

**p < 0.01 (asymptotic, two-tailed).

Particularly, the differences in the prescription rates of SGA depot preparations could be mainly attributed to significantly higher prescription rates of aripiprazole depot in women, which was not prescribed to men at all (see Table 6). On the other hand, men were significantly more frequently prescribed clozapine as an orally administered SGA. With regard to antidepressants, only venlafaxine had significantly higher prescription rates in men. However, none of these differences persisted after Bonferroni correction, so these results should be interpreted cautiously. No significant differences could be found for other specific antipsychotics, benzodiazepines, or anticonvulsants.

**TABLE 6:** Prescription rates of drugs frequently prescribed in male and female in-patients with schizophrenia.

	Men (*n* = 29)	Women (*n* = 29)		
		
	Prevalence in %	Prevalence in %	χ^2^	Significance level (*p*)
***Antidepressants***				
Venlafaxine	13.8%	0.0%	4.296	0.038*
***Second-generation antipsychotics***				
Clozapine	20.7%	3.4%	4.062	0.044*
Aripiprazole depot	0.0%	24.1%	7.961	0.005**

*χ^2^, Chi-square (Pearson).*

*p < 0.05, **p < 0.01 (asymptotic, two-tailed).

## Discussion

This study, for the first time, examined gender differences in the psychopharmacological treatment of forensic in-patients with schizophrenia. For this purpose, 29 female patients with schizophrenic disorders being treated in forensic psychiatry were compared with respective 29 male patients regarding demographic, clinical, and criminal data, psychopharmacological treatment strategies, psychopathological characteristics, as well as neurological and metabolic side effects.

The study was able to show that women with schizophrenia in forensic psychiatry differ from men in terms of sociodemographic characteristics quite remarkably, in that they have a higher level of education overall and are more likely to having entered into marriage prior to hospitalization. This corresponds not only with research on general psychiatric patients with schizophrenia (14), but also with results of a Swiss study on gender-specific differences in forensic patients with a schizophrenic disorder (12). Moreover, we were able confirm the finding that women treated in forensic psychiatry were more often separated from their spouses or divorced, which also was described earlier for female offenders with mental illnesses (28). In accordance with Günther et al. (12), we therefore argue that more attention should be paid to the ability to maintain relationships when treating female offenders with schizophrenia.

An interesting result of the present study is that men were more likely to have completed their occupational training, while women had an overall higher level of education. Since the onset of the disorder, objectified by the age at first hospitalization, showed no gender differences in contrast to previous studies with general psychiatric (14) as well as forensic samples (12), it can be assumed that the sampled women in this study experienced the onset of the disorder before graduating from college or university, leading to the typical sudden drop in social functioning and performance often accompanying schizophrenia onset (29), while men were able to complete occupational training on a lower educational level before falling ill. Thus, the integration in a fitting occupational setting as a treatment goal can be more challenging for women, which should carefully be considered when planning treatment or discharge.

Further discrepancies to earlier studies also arise from the fact that our forensic samples did not differ with regard to clinical characteristics such as number of prior hospitalizations, duration of illness, past substance abuse, and comorbidities. While women usually have more comorbid disorders (16), men experience longer durations of illness and more frequent hospitalizations (12, 14). In addition, prior studies in general psychiatric (13) as well as forensic (12) contexts showed men to be more likely to abuse alcohol and other substances. In line with Günther et al. (12), however, no differences could be found in the present study with regard to suicidality, self-harm, and a comorbid diagnosis of a personality disorder. In summary, our results suggest that female schizophrenia in-patients in forensic psychiatry are quite similar to men when it comes to clinical characteristics, with one exception: men reported schizophrenia in their family history more often than women. While information on family histories in forensic settings is still scarce (8), our findings support recent evidence for an increased family burden of mental illness among men with schizophrenia treated in forensic psychiatry (30).

Regarding criminal aspects, our findings correspond with previous research in that women were older when first incarcerated (12), committed arson more frequently (31), and engaged in fewer property and traffic offenses than men (12). Interestingly, women more often were found to be under the influence of a substance when committing the offense leading to hospitalization, supporting previous studies with mentally ill female offenders (28). Contrary to Günther et al. (12), as well as Wang et al. (30), the prevalence of violent offenses did not differ between genders. However, it must be noted that we rated all acts of violence as violent offenses, while Wang et al. (30) as well as Günther et al. (12) specifically analyzed gender differences with respect to capital offenses. Thus, as our findings are preliminary and need to be confirmed, we recommend a more detailed analysis of different violent offenses in more comprehensive studies in the future.

Concerning psychopharmacological treatment strategies, we found no differences between men and women for the potency of the prescribed doses, confirming previous findings in a forensic setting (12). However, lower doses are usually recommended for women due to differences in absorption and metabolism (16). Moreover, Bauer and Knörnschild (17) point out that dosage regimen should consider changes in estrogen levels and be adjusted according to the individual menopausal status, with young premenopausal women requiring lower doses and postmenopausal women requiring higher doses. However, we could not find differences in the potency of the dosages when stratifying for menopause in our female sample, suggesting a possible disregard for gender-specific dosage recommendations in clinical practice. In light of our results, we propose a more nuanced approach to the dosage of antipsychotic medication according to menopausal status. Referring to the neuroprotective effects of additional treatment with estrogen, especially in the case of very severe disease progression after menopause (17), Günther et al. (12) accordingly argue for lower doses for women in forensic treatment as standard doses are associated with overdoses and consequently with more side effects. Concerning neurological side effects, we indeed found more pronounced neurological impairments in the face and oral area in women, indicating possible overdosage. The fact that we found negative symptoms secondary to drug effects more pronounced in women could be interpreted as further evidence to that effect. However, it should be noted that women are generally more frequently affected by extrapyramidal side effects than men (17). Differences in the prescription rates of specific antipsychotics were found with regard to the more frequent use of clozapine in men and depot SGAs, primarily aripiprazole depot, in women. However, the latter may also be due to the fact that the depot formulation has not been approved in Germany until November 2013 (32), while data collection for men took place in 2014, making it a way more established antipsychotic when the female sample was surveyed in 2018 and 2019.

Differences in the metabolic side effects were found with regard to higher lipid levels in men. In line with Pillinger et al. (33), higher lipid levels could also be associated with more frequent use of clozapine, especially since Vasic et al. (8) found both significantly higher clozapine prescription rates and a trend for higher cholesterol levels in the forensic sample. Lower clozapine prescription rates in women found in this study could also be attributed to the more pronounced metabolic side effects of clozapine for women in general (13, 17, 34), making it an antipsychotic indicated for women in singular cases of treatment resistance.

Compared to men, women showed significantly worse psychopathological characteristics exhibiting more pronounced or less remitted symptoms than men, especially concerning their general psychopathology. While these findings contradict previous research in non-forensic settings (14), Günther et al. (12) also showed that women benefit less from in-patient forensic treatment than men when it comes to reducing psychopathological symptoms. Moreover, Tang et al. (15) also showed in a Chinese sample more severe and persistent positive and affective symptoms in women with schizophrenia. In contrast to previous findings (12, 14, 15) no gender differences in the onset of schizophrenia were found in this study, indicating a relatively early onset compared to other female samples. Earlier onset of schizophrenia usually means a more severe course of illness with long-lasting symptoms being relatively non-responsive to antipsychotic medication (16), which might provide an explanation as to why women showed worse psychopathology than men in this study. Since the treatment goal in forensic psychiatry pertains to the prevention of future delinquency through remission of psychopathology (7), a future research project could examine the influence of gender differences in the remission of psychopathological symptoms on delinquent recidivism. Moreover, women did not only show a worse response rate for single positive and negative symptoms, but also seemed to experience more pronounced feelings of guilt, anxiety, somatic discomfort, and depression, as well as less cognitive flexibility compared to men, indicating that female forensic patients with schizophrenia need more comprehensive treatment strategies and goals beyond the reduction of negative and positive symptoms. This is reflected in substantiated findings that women with schizophrenia (35) as well as female offenders with severe mental disorders (36) report more traumatic experiences then men and tend to act against their close family members (30). Landgraf et al. (37) showed in a pioneer study on clinical and demographic differences between forensic (*n* = 35) and general psychiatric (*n* = 35) female patients with schizophrenia that criminal behavior is associated with greater clinical impairment, higher rates of comorbidities, and suicidal behavior as well as worse socio-economic backgrounds. Moreover, a recent study showed violent victimization to be a better predictor for violent behavior than current psychopathology (38). Overall, the results of the present study suggest that female forensic patients with schizophrenia have more severe, clinically complex, and refractory diseases than their male counterparts.

When interpreting the results of our study, a number of limitations have to be considered. First, the data was collected by institutional personnel and therefore might be less reliable and valid compared to a standardized data collection done by non-institutional personnel. However, data quality for both samples was comparable thanks to the use of a standardized data entry form and collection procedure. Second, our findings are preliminary due to small sample sizes and the exploratory nature of this study. Nevertheless, some significant results persisted even after statistical correction for multiple testing, suggesting robust gender differences in psychopathological, neurological, and metabolic status. It must also be considered that our samples are subject to local limitations with the female sample being collected at a single institution and the male sample in two neighboring forensic institutions in the same federal state in Germany as the female sample (8). This makes it difficult to generalize and transfer the results to other institutions and regions, as the results could to some extent reflect local or clinic-specific treatment strategies. On the other hand, this unity of place can be seen as a methodological advantage in that it enabled homogeneous data collection and largely excluded other influencing factors such as regional or national differences in application practices (7).

Furthermore, as already mentioned, the more frequent use of SGAs in depot form in women could be based on a confounding cohort effect (39) due to the time lapse of 4–5 years between data collection for the male and female samples, making it difficult to clearly interpret the results. At the same time, this may counteract the disadvantages of a pure cross-sectional study and enable causal inferences regarding the relationship between psychopharmacological treatment and the occurrence of side effects (8). Thus, one could hypothesize that, in the sense of a trend to evaluate antipsychotic drugs based on the side effect profiles in accordance with Hasan et al. (1), the prescription of the novel aripiprazole depot preparation in the female sample occurred in favor of the goal of reducing metabolic side effects (40), albeit at the expense of residual psychopathological symptoms. This hypothesis could be tested in a future study, for example by questioning the treating physicians. Further research with larger samples and several locations should also follow in order to substantiate our findings and examine gender-specific differences in psychopharmacological treatment strategies with regard to recidivism.

## Data Availability Statement

The raw data supporting the conclusions of this article will be made available by the authors, without undue reservation.

## Ethics Statement

The studies involving human participants were reviewed and approved by the Ethics Committee of Ulm University. The patients/participants provided their written informed consent to participate in this study.

## Author Contributions

MD, VK, PR, and NV designed the study. JM, VW, IS, PR, and MF were responsible for administration of data collection. JM, PR, and JS conducted the literature research. JM wrote the first draft of the manuscript. JM and JS conducted the statistical analysis. All authors read and approved the final version of the manuscript.

## Conflict of Interest

The authors declare that the research was conducted in the absence of any commercial or financial relationships that could be construed as a potential conflict of interest.

## Publisher’s Note

All claims expressed in this article are solely those of the authors and do not necessarily represent those of their affiliated organizations, or those of the publisher, the editors and the reviewers. Any product that may be evaluated in this article, or claim that may be made by its manufacturer, is not guaranteed or endorsed by the publisher.
